# Neck-cooling improves repeated sprint performance in the heat

**DOI:** 10.3389/fphys.2015.00314

**Published:** 2015-11-05

**Authors:** Caroline Sunderland, Ryan Stevens, Bethan Everson, Christopher J. Tyler

**Affiliations:** ^1^Department of Sports Science, School of Science and Technology, Sport, Health and Performance Enhancement Research Centre, Nottingham Trent UniversityNottingham, UK; ^2^Department of Life Sciences, University of RoehamptonLondon, UK

**Keywords:** hyperthermia, team-sport, high-intensity, thermoregulation, treadmill, pacing

## Abstract

The present study evaluated the effect of neck-cooling during exercise on repeated sprint ability in a hot environment. Seven team-sport playing males completed two experimental trials involving repeated sprint exercise (5 × 6 s) before and after two 45 min bouts of a football specific intermittent treadmill protocol in the heat (33.0 ± 0.2°C; 53 ± 2% relative humidity). Participants wore a neck-cooling collar in one of the trials (CC). Mean power output and peak power output declined over time in both trials but were higher in CC (540 ± 99 v 507 ± 122 W, *d* = 0.32; 719 ± 158 v 680 ± 182 W, *d* = 0.24 respectively). The improved power output was particularly pronounced (*d* = 0.51–0.88) after the 2nd 45 min bout but the CC had no effect on % fatigue. The collar lowered neck temperature and the thermal sensation of the neck (*P* < 0.001) but had no effect on heart rate, fluid loss, fluid consumption, lactate, glucose, plasma volume change, cortisol, or thermal sensation (*P* > 0.05). There were no trial differences but interaction effects were demonstrated for prolactin concentration and rating of perceived exertion (RPE). Prolactin concentration was initially higher in the collar cold trial and then was lower from 45 min onwards (interaction trial × time *P* = 0.04). RPE was lower during the football intermittent treadmill protocol in the collar cold trial (interaction trial × time *P* = 0.01). Neck-cooling during exercise improves repeated sprint performance in a hot environment without altering physiological or neuroendocrinological responses. RPE is reduced and may partially explain the performance improvement.

## Introduction

Continuous and intermittent exercise performance are impaired when ambient temperatures are elevated (Drust et al., 2005; Morris et al., 2005; Tyler and Sunderland, 2008). One countermeasure is the application of cooling either before (pre-cooling) or during exercise (Bongers et al., 2014; Tyler et al., 2015). Although, pre-cooling is the more common approach, interestingly cooling during exercise offers a similar magnitude of benefit to exercise performance in hot conditions, and both approaches enhance exercise performance with and without physiological alterations (Bongers et al., 2014; Tyler et al., 2015). The fact that a cooling-induced physiological change, such as a lowered deep body temperature, is not required to improve exercise performance in the heat, is of particular importance. Although, it is well-established that aggressive, high-powered cooling interventions such as water-immersion are the most effective for cooling the body, these traditional cooling interventions are cumbersome with limited “real world” application especially during activity.

There are some recent data on internal cooling interventions such as the ingestion of ice-slurries (Siegel et al., 2010; Siegel and Laursen, 2012); however, external cooling interventions such as vests and neck-cooling collars have been more extensively researched. The majority of the data focusses on pre-cooling but recent data have shown that cooling the torso and neck during exercise can improve capacity and performance in the heat (Tyler et al., 2010; Kenny et al., 2011; Tyler and Sunderland, 2011a,b). Cooling individuals during exercise could be especially attractive for team-sports such as soccer, rugby and field hockey which regularly take-place in hot conditions and could utilize a cooling intervention during training, warm-up bouts, active half-time interventions, and in the case of sports such as field hockey, between rolling-substitution cycles (Arngrïmsson et al., 2004). Previous data suggest that the neck region may be an effective site to cool due to practical considerations (e.g., in most sports the neck is more readily accessible than the torso) as well as anatomical and perceptual ones (e.g., the neck is in close proximity to the thermoregulatory center and a region of high alliesthesial thermosensitivity, Cotter and Taylor, 2005). Previous data reported that cooling the neck is more effective than cooling the same surface area of the trunk in the alleviation of heat strain (Shvartz, 1976) but neck-cooling during exercise does not appear to have any measurable effect on the peripheral physiological or biochemical response to prolonged steady-state exercise in the heat (Gordon et al., 1990; Bulbulian et al., 1999; Tyler et al., 2010; Tyler and Sunderland, 2011a,b; Lee et al., 2014). Despite the lack of physiological change, neck-cooling collars worn during exercise have been shown to enhance 15 min preloaded treadmill time-trial performance (closed-loop test) by ~6% (*d* = 0.29–0.67) (Tyler et al., 2010; Tyler and Sunderland, 2011b) and treadmill running capacity (open-loop test) by ~13% (*d* = 0.43) (Tyler and Sunderland, 2011a) and ~6% (*d* = 0.63) (Lee et al., 2014) in hot environmental conditions (30–32°C; 50–70% rh).

Intermittent-exercise bouts of varying intensity are common in team-sports and such exercise places different stressors upon the body than steady-state exercise. In cool conditions, the different exercise types elicit similar physiological responses (Drust et al., 2000); however, in hot conditions intermittent exercise increases the thermoregulatory, cardiovascular and metabolic stresses compared to continuous exercise (Ekblom et al., 1971; Mora-Rodriguez et al., 2008). Success in intermittent sports is heavily linked to the ability to perform repeated bouts of high-intensity sprint exercise but this ability is impaired in elevated temperatures and this impairment is particularly pronounced during the latter stages of a match (Mohr et al., 2010). During high-intensity intermittent running in the heat there is a rapid rise in deep body temperature (Sunderland et al., 2008), a greater thermal strain experienced, and a decrease in skill performance (Sunderland and Nevill, 2005). A potential cause for the decreased repeated sprint performance observed during team sport activity in the heat is an elevated core and brain temperature, and resultant impairment of central nervous system function (Girard et al., 2015). It has been suggested that the temperature-sensitive areas of the hypothalamus release inhibitory signals due to the high brain temperature which impairs motor activity (Nybo, 2012) and alters neurotransmitter release (Meeusen and Roelands, 2010). Neck cooling is unlikely to reduce human brain temperature during exercise (Sukstanskii and Yablonskiy, 2007; Nybo et al., 2014) and has no effect on core body temperature (Tyler et al., 2010; Tyler and Sunderland, 2011a,b); however, it can dampen the perceived magnitude of thermal strain allowing individuals to tolerate higher core body temperatures and heart rates before volitional termination (Tyler and Sunderland, 2011a). The effectiveness of such a dampening effect appears to be dependent upon the magnitude of thermal strain experienced (Tyler et al., 2010) and so cooling the neck may be beneficial during intermittent activity in the heat when core (Sunderland and Nevill, 2003, 2005; Morris et al., 2005; Sunderland et al., 2008), and thus brain temperatures, can exceed 39.5°C.

Cooling prior to intermittent activity has been reported to improve subsequent performance in some but not all cases and overall offers a small beneficial effect (mean *d* = 0.44) to such activity (Tyler et al., 2015). To date, cooling during exercise has only been investigated during prolonged, steady-state exercise in the heat; however, intermittent exercise performance is also impaired in the heat (Ekblom et al., 1971; Morris et al., 2005; Mora-Rodriguez et al., 2008). High-intensity, intermittent activity might place a greater thermal strain on the body than steady-state exercise and so it is prudent to suggest that neck-cooling might be more beneficial to such activity if the perceived level of strain is dampened. The aim of the current study was to address the gap in the literature and investigate the effect of cooling the neck region on repeated sprint performance and the physiological and neuroendocrinological responses to such activity in the heat. We hypothesized that neck cooling would enhance repeated sprint performance and thermal comfort during intermittent running in the heat.

## Materials and methods

### Participants

Seven recreationally active, non-heat acclimated male games players (age: 25.9 ± 3.4 years, height: 180.9 ± 6.1 cm, mass: 81.2 ± 8.2 kg and maximal oxygen uptake ($\dot{\text{V}}$O_2max_): 53.5 ± 2.7 ml·kg^−1^·min^−1^) volunteered for the study. All participants engaged in at least 3–4 h of team sports per week (2 were semi-professional) and had not been exposed to high ambient temperatures for 2 months prior to the investigation. Participants provided written informed consent to participate in the study and were free from illness and injury determined by a health screening questionnaire. Twenty four hour before the first trial, a food diary was completed and replicated prior to any further trials. Participants arrived to the laboratory 2 h fasted and abstained from alcohol, caffeine, and strenuous exercise 24 h before testing. All trials took place during winter and early spring with mean temperatures ranging from 2.2 to 10.2°C. This study was approved by the Nottingham Trent University's Ethical Advisory Committee.

### Experimental design

Participants completed a preliminary visit [to measure anthropometric measurements and $\dot{\text{V}}$O_2max:_ using a maximal incremental exercise test (details below)] and one familiarization trial before completing two experimental trials in a randomized (via lot draw) cross-over design. Each session was separated by 7 days and completed at the same time of day to control for the effect of circadian rhythm on body temperature (Reilly and Brooks, 1986). In one experimental trial participants wore the neck-cooling collar (CC trial) for the entire duration and for the other visit, no cooling collar (NC trial) was worn. All trials took place in a walk-in environmental chamber (model WIR52-20HS; Design Environmental LTD, Gwent, United Kingdom) in hot ambient conditions (33.0 ± 0.2°C; 53% ± 2% relative humidity). The familiarization, CC and NC trials involved participants completing the following tests: 5 × 6 s sprints and a football specific intermittent treadmill protocol (FSINT). During CC and NC, repeated sprints were completed pre FSINT (Set 1), at half-time (Set 2) and post FSINT (Set 3). However during the familiarization trial sprints were conducted pre (Set 1) and post (Set 2) a 45 min of the FSINT1.

### Experimental procedures

#### Speed lactate and maximal oxygen uptake test

Participants began an incremental running speed lactate test at a self-selected starting speed (range: 6–10 km·h^−1^) on a motorized treadmill (Pulsar, h/p/cosmos, Germany). Exercise intensity was increased by 1 km·h^−1^ every 3 min until an increase of lactate above 4 mmol· L^−1^ was reached. Capillary blood samples were obtained from the finger at the end of every stage and analyzed for lactate using an automated glucose and lactate analyser (YSI 2300 Stat, YSI Incorporated, USA). Participants rested for 10 min before performing a $\dot{\text{V}}$O_2max_ test to volitional exhaustion. Treadmill speed was equivalent to that of individual lactate threshold, and began at a 1% incline. The gradient was increased by 1% every minute until the participant indicated they had only 1 min remaining (Jones and Doust, 1996). A Douglas bag sample was obtained during the final min and analyzed for gas content and volume using a calibrated Servomex gas analyser (Servomex, United Kingdom) and dry gas meter (Harvard, United Kingdom).

#### Football specific intermittent treadmill protocol (FSINT)

Participants completed a laboratory based intermittent treadmill protocol designed to replicate the demands of football (Saunders et al., 2014). The protocol consisted of two 45 min halves (FSINT1 and FSINT2) separated by a 15 min half time period. During each half, the protocol comprised of three 15 min activity bouts consisting of alternating exercise intensities performed on a motorized treadmill (Pulsar, h/p/cosmos, Germany) set at a 1% gradient. Total distance covered during the FSINT was 9.72 km.

#### Repeated sprints (5 × 6 s)

The repeated sprints protocol consisted of five, 6 s maximal sprints on a non-motorized treadmill, with each sprint separated by a 24 s active recovery (Desmo-Force, Woodway, USA). Data was recorded at 200 Hz and averaged over a rolling 1 s. Data from this laboratory has previously shown that the coefficient of variation of the 5 × 6 s sprint test is 2.11% for mean power output (MPO) and 2.34% for peak power output (PPO) (Saunders et al., 2014). When sprinting, participants were required to wear a belt around their waist which was attached to a force transducer placed directly behind the treadmill. Participants were instructed to perform each sprint maximally, and were given strong verbal encouragement for the duration of every sprint. All data were recorded using a modified version of Spike2 (V5.09, CED, Cambridge, United Kingdom).

The 5 × 6 s repeated sprint protocol was performed on three occasions; following a standardized 5 min warm up comprising running at 10 km·h^−1^ (Set 1), immediately following FSINT1 (Set 2) and immediately following the FSINT2 (Set 3). MPO and PPO of every sprint were recorded; MPO was determined as the highest average power output over 6 s for each sprint. Percentage fatigue for MPO and PPO during each set of the sprint protocol was calculated using the following equation and following recommendations which showed the performance decrement score to be the most suitable formula for determining fatigue during repeated sprint exercise (Glaister et al., 2008):

% fatigue = 100−([total power output/ideal power output] × 100),

where total power output represents the sum of the power output values for all sprints during the set, and ideal power output represents the number of sprints performed multiplied by the highest power output of all sprints in the set.

#### Cooling collar

In the CC trial participants wore a modified commercially available neck-cooling collar (model CCX; Black Ice LLC, Lakeland, USA) as used in previous research (Tyler et al., 2010; Tyler and Sunderland, 2011a,b; Lee et al., 2014). The CC was adapted by draining the inner 5 compartments of the Black Ice cooling reagent and filling it with approximately 120 g of gel refrigerant (BDH Laboratory Supplies, Poole, United Kingdom) in order to provide the greatest magnitude of cooling without causing tissue damage (unpublished observations). The cooling part of the collar was held in place by a 600 mm neoprene wrap with a hook and loop to fasten around the neck. At room temperature the collar weighed 155 g and the dimensions were length 375 mm × width 60 mm, and depth 15 mm. 24 h prior to the trial the collar was placed into a freezer at −80°C. 10 min before application, the CC was placed in an ambient temperature to clear any surface frost. The collar was placed on the participant immediately prior to entry into the chamber.

#### Total fluid consumption and body mass

Participants were required to consume 500 ml of water 2 h prior to the trial in an attempt to ensure euhydration. During all trials, participants consumed water (30°C ± 2.1°C) *ad libitum* and the volume was recorded. Nude body mass was recorded (Adam CFW 150 scale; Adam Equipment Co Ltd, Milton Keynes, United Kingdom) pre and post experimental trials for the estimation of sweat loss.

#### Thermistors and rectal temperature (tre)/heart rate monitoring

Participants self-inserted a rectal probe (model REC-U-VL-0; Grant Instruments [Cambridge] Ltd, Cambridgeshire, United Kingdom) approximately 10 cm past the anal sphincter. Neck thermistors (model EUS-U-VL-3; Grant Instruments [Cambridge] Ltd, Cambridgeshire, United Kingdom) were attached across the posterior aspect of the neck with waterproof tape (Transpore; 3M, St Paul, MN, USA) and transparent dressing to stabilize the thermistors (Tegaderm; 3M, St Paul, MN, USA). Additionally, 4 thermistors were attached on the center of the right pectoral muscle, and the belly of the following muscles, flexi carpi radials, gastrocnemius, and quadriceps muscle for the calculation of mean weighted skin temperature (Ramanathan, 1964). Participants also wore a heart rate (HR) monitor (model RS400; Polar Electro Oy, Kemple, Finland) that was attached before entering the environmental chamber. HR, mean neck skin temperature, weighted-mean skin temperature, and T_re_ were all measured in 15 min intervals for the duration of CC and NC.

#### Collection and analysis of blood samples

Venous blood samples were taken in a standing position, at baseline in ambient conditions and immediately prior to and immediately following every sprint bout in the heat, resulting in seven individual samples (Baseline, Pre-exercise, Post Set 1, Post FSINT 1, Post Set 2, Post FSINT 2, Post Set 3) at which times participants were stationary. Whole blood was initially analyzed for lactate and glucose (Yellow Springs Instrument, 2300 STAT plus, Yellow Springs Instruments Inc., USA) and then aliquots were dispensed into K_3_-EDTA, LH and serum tubes (Sarstedt Ltd, Leicester, United Kingdom). The aliquots were then centrifuged at 4000 g for 10 min at 4°C. After centrifuging the supernatant was removed and then frozen at −80°C until the analyses were performed.

Changes in blood, plasma and red cell volume were calculated from the mean hemoglobin concentration (cyanomethaemoglobin method, Cecil Instruments, Cambridge, United Kingdom, measured in duplicate) and the mean haematocrit (Micocentrifugation, Hawksley, Sussex, United Kingdom, in triplicate) using the standard equations (Dill and Costill, 1974).

Serum concentrations of cortisol and plasma prolactin were determined via enzyme-linked immunosorbent assays (R&D Systems, Abingdon, United Kingdom). The intra-assay coefficient of variation for the cortisol and prolactin ELISAs were 5.4 and 7.7% respectively.

#### Perceptual variables

Rating of perceived exertion (RPE) (Borg, 1982), neck thermal sensation (TS_neck_) and whole-body thermal sensation (TS) (Young et al., 1987) were taken at 15 min intervals during experimental trials. A nine-point scale, ranging from 0 (unbearably cold) to 8 (unbearably hot) with 4 as comfortable (neutral) was used to measure thermal sensation and thermal sensation of the neck_._ Participants were instructed to differentiate between neck thermal sensation and thermal sensationof the body.

#### Statistical analyses

Descriptive data are reported as mean ± standard deviation and data were checked for normality. Three-way factorial ANOVA [trial (2 levels) × set (3 levels) × sprint (5 levels)] was used to determine any difference in power output while two-way (trial × time) tests were performed to evaluate differences between trials for, thermoregulatory, cardiovascular, neuroendocrinological, and perceptual variables. Paired *t*-tests were conducted to evaluate differences between sweat-loss, fluid consumption and changes observed over the course of the trial. Following a significant *F*-value Tukey's HSD *post hoc* tests were conducted to identify pair-wise differences. Violations of sphericity were adjusted for using the Greenhouse Geisser adjustment when appropriate. The effect size (Cohen's *d*) of all significant differences was calculated using trial pairings and interpreted using the following thresholds: < 0.2 = trivial effect; 0.2–0.5 = small effect; 0.5–0.8 = moderate effect and >0.8 = large effect (Cohen, 1992). *A priori* power analysis (β = 0.2) was completed on the primary outcome variable of power output which estimated a sample size of 7 would be sufficient to detect a difference. Significance was set a priori at the *P* < 0.05 level.

## Results

### Neck temperature

**Figure 1:**
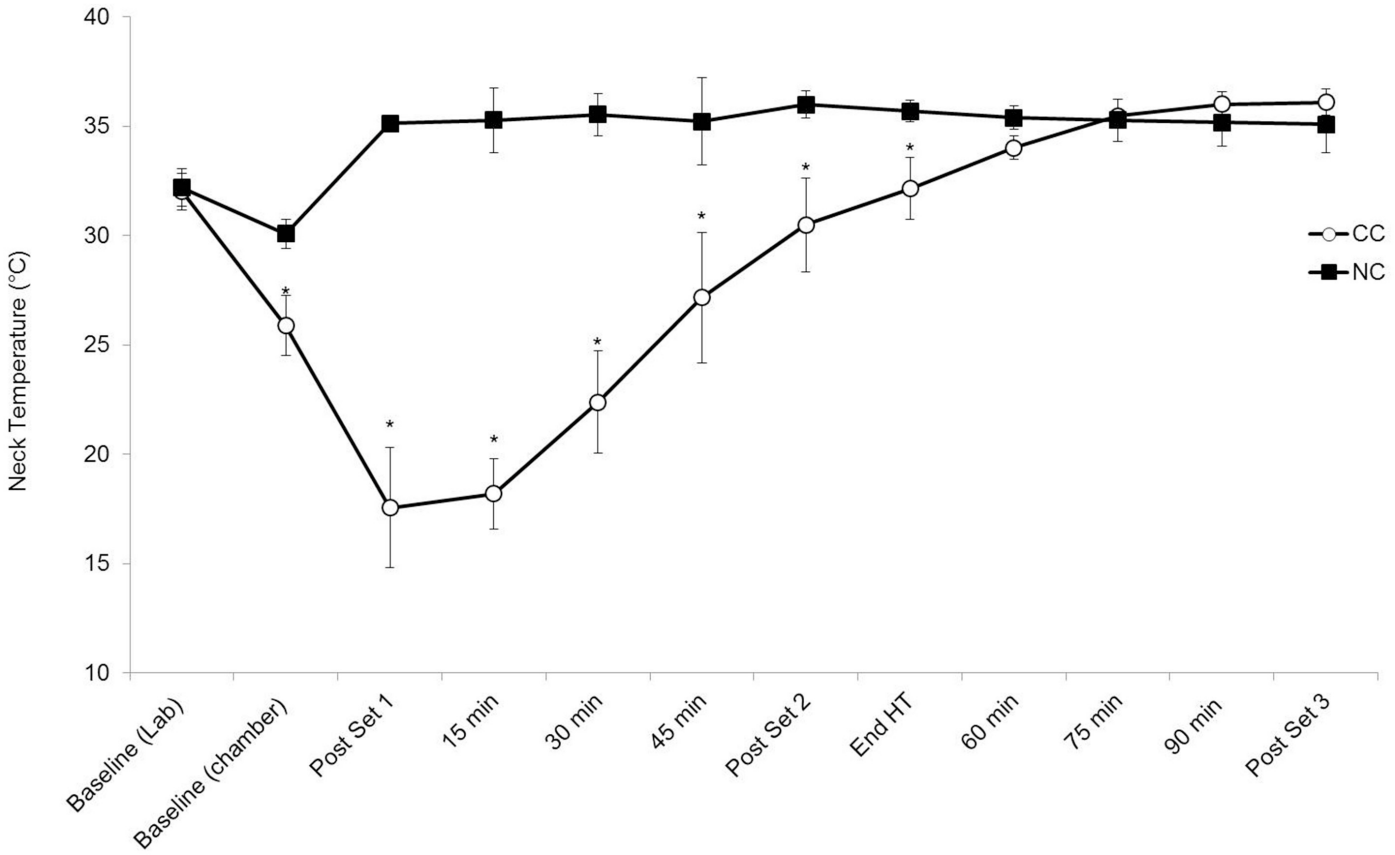
**The mean (±1 SD) neck skin temperature**. Main effect trial (*P* < 0.001), time (*P* < 0.001) and interaction (*P* < 0.001). ^**^P < 0.01.

Mean neck skin temperature is shown in Figure 1. Neck temperature was lower during CC than NC [trial *P* < 0.001, *d* = 1.27 (large effect)] and changed with time (*P* < 0.001). After application of the cooling collar neck temperature remained lower for CC than NC until 60 min (interaction trial × time *P* < 0.001, *post hoc P* < 0.05).

### 5 × 6 s sprint performance

**Figure 2:**
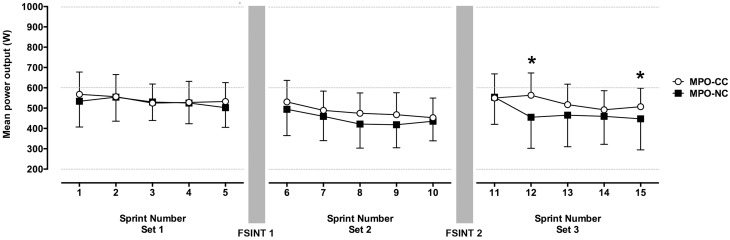
**The mean (±1 SD) mean power outputs (MPO) during the 15.6 s sprints before and after 2 bouts of soccer-specific intermittent exercise with and without a cold collar**. Main effect trial (*P* = 0.01, *d* = 0.32), interaction trial × set × sprint (*P* = 0.0001). ^*^P < 0.05.

MPO was greater during CC (539.9 ± 98.8 W) than NC [506.7 ± 121.7 W; trial *P* = 0.01; *d* = 0.32 (small effect)], with a decline in MPO with increasing number of sprints performed (sprint *P* = 0.003; Figure 2). There was an interaction effect (trial × set × sprint *P* = 0.0001) which indicated greater MPO during the CC particularly after FSINT 2, when performance was improved for sprints 12 [*post hoc P* < 0.001; *d* = 0.88 (large effect)] and 15 [*post hoc P* < 0.05; *d* = 0.51 (moderate effect); Figure 2].

There was no effect of neck cooling on % fatigue (trial P = 0.55), though fatigue increased with number of sets completed (set P = 0.02). Fatigue was 7.6 ± 4.3, 11.7 ± 7.3, and 13.1 ± 9.4% for set 1, 2, and 3 respectively.

**Figure 3:**
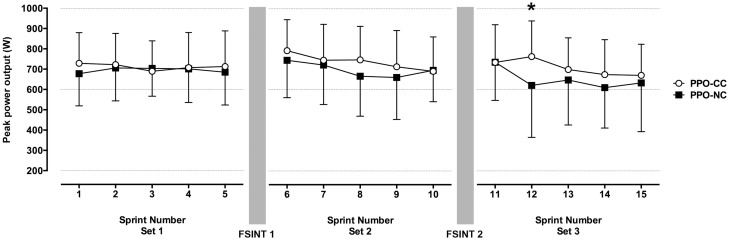
**The mean (±1 SD) peak power outputs (PPO) during the 15.6 s sprints before and after 2 bouts of soccer-specific intermittent exercise with and without a cold collar**. Main effect trial (*P* = 0.03, *d* = 0.24), interaction trial × set × sprint (*P* = 0.002). ^*^P < 0.001.

PPO was greater during CC (718.8 ± 158.0 W) than NC [680.2 ± 182.1 W; trial P = 0.03; *d* = 0.24 (small effect)], with a decline in PPO with increasing number of sprints performed (sprint *P* < 0.05; Figure 3). There was an interaction effect (trial × set × sprint *P* = 0.002) which indicated greater PPO during the CC particularly after FSINT 2, when performance was improved for sprint 12 [*post hoc P* < 0.001; *d* = 0.70 (moderate effect); Figure 3].

### Heart rate, rectal temperature and mean skin temperature

*T*_re_ data is presented for *n* = 6 due to equipment malfunction during one of the trials. Heart rate and *T*_re_ increased significantly over the duration of the trial (main effect time HR and *T*_re_: *P* < 0.001); however, there were no significant differences between trials for *T*_re_ [trial *P* = 0.15, *d* = 0.32 (small effect)] or HR [trial *P* = 0.23, *d* = 0.06 (trivial effect)]. There were no significant differences between trials in the changes observed across the entire exercise period for *T*_re_ (trial *P* = 0.89) or HR (trial *P* = 0.95). The *T*_re_, mean skin temperature and HR data observed at baseline, after each set of sprints and at 45 and 90 min are shown in Table 1. Mean skin temperature was not different between the no collar and cold collar conditions (trial *P* = 0.52, *d* = 0.07; interaction trial × time *P* = 0.60), and increased with exercise duration (time *P* < 0.0001).

**Table 1:** **Mean ± SD rectal temperature, skin temperature and heart rate during the intermittent running**.

	**Baseline**	**Post set 1**	**Post FSINT 1 (45 min)**	**Post set 2**	**Post FSINT 2 (90 min)**	**Post Set 3**	**Δtrial**
**RECTAL TEMPERATURE (°C)**							
NC	37.0 ± 0.3	37.1 ± 0.3	38.4 ± 0.3	38.6 ± 0.3	38.8 ± 0.4	38.9 ± 0.4	1.9 ± 0.5
CC	36.9 ± 0.2	36.9 ± 0.2	38.2 ± 0.2	38.3 ± 0.2	38.6 ± 0.3	38.7 ± 0.4	1.8 ± 0.4
Mean difference (95% CI)	0.1 (−0.3, 0.5)	0.2 (−0.2, 0.6)	0.2 (0.0, 0.4)	0.3 (0.0, 0.5)	0.2 (0.0, 0.5)	0.3 (0.0, 0.6)	
	Trial *P* = 0.15, time *P* < 0.0001 and interaction trial x time *P* = 0.89.						
**MEAN SKIN TEMPERATURE (°C)**							
NC	33.2 ± 0.7	34.0 ± 0.6	35.2 ± 1.2	34.6 ± 1.9	35.1 ± 1.4	35.1 ± 1.5	1.8 ± 1.3
CC	32.9 ± 0.5	33.9 ± 0.7	35.2 ± 1.0	35.4 ± 0.8	35.2 ± 1.2	34.8 ± 1.8	1.8 ± 1.6
Mean difference (95% CI)	0.3 (−0.1, 0.6)	0.1 (−0.2, 0.3)	0.1 (−1.0, 1.1)	−0.8 (−2.3, 0.7)	−0.1 (−1.1, 0.8)	0.3 (−1.1, 1.6)	
	Trial *P* = 0.52, time *P* < 0.0001 and interaction trial x time *P* = 0.60.						
**HEART RATE (BEATS·MIN^−1^)**							
NC	68 ± 17	152 ± 12	140 ± 10	165 ± 10	149 ± 19	172 ± 9	105 ± 18
CC	65 ± 12	153 ± 9	136 ± 11	166 ± 9	144 ± 11	170 ± 12	106 ± 10
Mean difference (95% CI)	3 (−9, 15)	−1 (−6, 4)	4 (−9, 17)	−1 (−4, 2)	5 (−8, 18)	2 (−2, 6)	
	Trial *P* = 0.23, time *P* < 0.0001 and interaction trial x time *P* = 0.95.						

*NC, no collar; CC, cold collar*.

### Perceptual measurements

All perceptual data (thermal sensation; thermal sensation of the neck; RPE) changed over time (main effect time all *P* < 0.001). There were no significant main trial or interaction effects for thermal sensation [*P* = 0.22, *d* = 0.2 (small effect), and *P* = 0.18 respectively; Table 2]. Thermal sensation of the neck was lower during CC than NC [trial *P* < 0.001, *d* = 1.34 (large effect)] and there was a significant interaction (trial × time *P* < 0.001; Table 2). RPE was not different between NC and CC [trial *P* = 0.07, *d* = 0.1 (trivial effect)], but did change differently within trials (interaction trial × time *P* = 0.01) with lower RPE during FSINT 1 and FSINT 2 during the CC trial.

**Table 2:** **Mean ± SD rating of perceived exertion, and thermal sensation of the neck and body during the intermittent running**.

	**Baseline**	**Post Set 1**	**Post FSINT 1 (45 min)**	**Post Set 2**	**Post FSINT 2 (90 min)**	**Post Set 3**	**Δtrial**
**RATING OF PERCEIVED EXERTION**							
NC	6.1 ± 0.4	15.7 ± 1.3	14.4 ± 1.6	17.6 ± 1.3	16.4 ± 0.8^*^	19.0 ± 1.0	12.9 ± 0.9
CC	6.1 ± 0.4	16.7 ± 1.5	13.6 ± 1.3	17.6 ± 1.0	15.6 ± 0.8	19.0 ± 0.8	12.9 ± 0.9
Mean difference (95% CI)	0.0 (0, 0)	−1.0 (−2.0, 0.0)	0.9 (−0.4, 2.2)	0.0 (−1.7, 1.7)	0.9 (0.0, 1.7)	0.0 (−0.8, 0.8)	
	Trial P = 0.07, time P = 0.0001 and interaction trial x time *P* = 0.01.						
**THERMAL SENSATION NECK**							
NC	4.0 ± 0.5	4.9 ± 0.7^**^	5.7 ± 1.2^**^	6.1 ± 1.1^**^	6.1 ± 1.1	6.6 ± 1.4	2.6 ± 1.4
CC	4.1 ± 0.5	1.9 ± 0.7	3.4 ± 0.3	3.6 ± 0.4	5.4 ± 0.8	6.1 ± 1.1	2.0 ± 0.9
Mean difference (95% CI)	−0.1 (−0.5, 0.2)	3.0 (1.7, 4.3)	2.3 (1.2, 3.7)	2.4 (1.2, 3.7)	0.7 (0.2, 1.3)	0.4 (0.0, 0.9)	
	Trial *P* < 0.001, time *P* < 0.0001 and interaction trial x time *P* < 0.0001.						
**THERMAL SENSATION BODY**							
NC	4.0 ± 0.6	5.2 ± 0.8	6.0 ± 1.0	6.4 ± 1.0	6.5 ± 1.2	6.9 ± 1.2	2.9 ± 1.5
CC	4.0 ± 0.0	5.1 ± 0.7	5.6 ± 0.9	6.1 ± 0.9	6.4 ± 1.0	6.8 ± 1.2	2.8 ± 1.2
Mean difference (95% CI)	0.0 (−0.6, 0.6)	0.1 (−0.6, 0.7)	0.4 (−0.3, 1.0)	0.2 (−0.3, 0.7)	0.1 (−0.4, 0.7)	0.1 (−0.1, 0.3)	
	Trial *P* = 0.22, time *P* < 0.0001 and interaction trial x time *P* = 0.18.						

NC, no collar; CC, cold collar;

** *= P < 0.01 from CC*,

* *P = 0.05 from CC*.

### Body fluid balance

There were no significant differences in the volume of water voluntarily consumed (*P* = 0.98) or in the volume of sweat lost (*P* = 0.99) between trials. Participants consumed 1.07 ± 0.25 L and 1.06 ± 0.60 L of water and lost 1.09 ± 0.43 L and 1.10 ± 0.71 L of sweat during the NC, and CC trials. The mean plasma volume changes observed was similar between trials (*P* = 0.64).

### Blood data

Whole blood lactate and glucose concentrations increased over time (both time *P* < 0.001), but there were no significant differences between trials for whole blood lactate [*P* = 0.87, *d* = 0.0 (no effect)] or glucose concentrations [*P* = 0.08, *d* = 0.42 (small effect)]. There was no significant increase in serum cortisol concentrations over time (*P* = 0.70) or significant differences between trials [*P* = 0.43, *d* = 0.23 (small effect); interaction trial × time *P* = 0.11; Table 3]. Plasma concentrations of prolactin were similar between trials (trial *P* = 0.75, *d* = 0.0 (no effect)], increased with time (*P* = 0.001) and showed a trial × time interaction effect (*P* = 0.04; Table 3). *Post-hoc* analysis did not reveal any pairwise differences.

**Table 3:** **Mean ± SD serum cortisol and plasma prolactin concentrations during the intermittent running**.

	**Baseline**	**Post set 1**	**Post FSINT 1 (45 min)**	**Post set 2**	**Post FSINT 2 (90 min)**	**Post set 3**
**CORTISOL (nmol·l^−1^)**						
NC	187.8 ± 82.4	150.4 ± 71.1	139.5 ± 37.6	159.8 ± 73.2	194.8 ± 91.8	212.6 ± 99.1
CC	203.1 ± 84.5	254.9 ± 150.7	144.3 ± 63.8	150.0 ± 60.2	190.4 ± 131.2	189.6 ± 148.0
Mean difference (95% CI)	−15.3 (−54.0, 23.5)	−104.5 (−214.9, 5.9)	−4.8 (−75.0, 65.4)	9.7 (−77.6, 97.0)	4.4 (−132.0, 140.9)	23.0 (−117.6, 163.5)
	Trial *P* = 0.431, time *P* = 0.698 and interaction trial x time *P* = 0.114.					
**PROLACTIN (nmol·l^−1^)**						
NC	0.56 ± 0.18	0.54 ± 0.22	1.00 ± 0.56	1.02 ± 0.59	1.48 ± 1.02	1.51 ± 1.08
CC	0.60 ± 0.28	0.62 ± 0.28	0.87 ± 0.35	0.93 ± 0.40	1.38 ± 1.02	1.45 ± 1.00
Mean difference (95% CI)	−0.04 (−0.28, 0.20)	−0.08 (−0.31, 0.16)	0.13 (−0.22, 0.48)	0.09 (−0.21, 0.39)	0.10 (−0.24, 0.44)	0.07 (−0.08, 0.21)
	Trial *P* = 0.747, time *P* < 0.001 and interaction trial x time *P* = 0.045.					

## Discussion

Intermittent exercise is impaired in hot, compared to moderate, conditions (Drust et al., 2005; Morris et al., 2005) and as a result researchers and practitioners are interested in interventions which may attenuate the impairment observed. The current study is the first to demonstrate that cooling the neck region during exercise can improve repeated sprint performance in the heat although % fatigue is unaffected. The present data builds upon previous data demonstrating that when exposed to hot conditions, neck-cooling during exercise can improve time-trial performance and exercise capacity (Tyler et al., 2010; Tyler and Sunderland, 2011a,b; Lee et al., 2014) as well as the performance of complex cognitive tasks (Lee et al., 2014) and so neck-cooling appears to be a potentially very useful intervention for team-sport players.

As previously reported, cooling the neck region lowered mean neck skin temperature without having an effect on HR, *T*_re_, mean skin temperature, volitional water consumption, sweat loss, or concentrations of lactate, glucose, cortisol, and prolactin (Tyler et al., 2010; Tyler and Sunderland, 2011a,b; Lee et al., 2014). Cooling-induced alterations in the physiological and hormonal responses to exercise in the heat appear to be dependent on the magnitude of cooling provided (Brisson et al., 1987; Tyler et al., 2015) and so it seems prudent to attribute the lack of effect to the low surface area cooled by the collar. Despite the lack of physiological or neuroendocrinological alterations MPO and PPO were both higher in the CC trial demonstrating that neck-cooling during exercise in the heat offers a benefit to repeated sprint ability. This benefit was of particular note in the latter stages of the trial. The small effect sizes observed when looking at overall MPO and PPO (*d* = 0.32 and 0.24 respectively) are comparable with some neck-cooling data (Tyler et al., 2010) (*d* = 0.23–0.29) but lower than others (Tyler and Sunderland, 2011a,b; Lee et al., 2014; *d* = 0.43–0.67) which were more comparable to the moderate-to-large effect sizes reported for the latter sprints in the current study (*d* = 0.51–0.88). Because the magnitude of cooling provided and the level of thermal strain experienced influence the effectiveness of a cooling intervention (Tyler et al., 2015) it is not unsurprising that the larger effect sizes are observed toward the end of the trial when the highest body temperatures are observed. The core temperatures observed at the end of the exercise bout in the present study are comparable with those observed in other neck-cooling investigations and so the lower effect sizes reported in the present study suggest that neck-cooling during exercise may be of greater benefit to more prolonged exercise but further data are required to confirm this.

Ansley et al. (2008) attributed an improved exercise capacity in the heat achieved by cooling the head during exercise in the absence of physiological changes to a centrally-mediated overriding of a “stop signal” and postulated that this was due to a reduction in brain temperature and an altered cerebral neuroendocrinological response. Animal data show that brain temperature appears to be the dominant regulator of exercise tolerance (Caputa et al., 1986) although for practical reasons human investigations have relied on core body temperature (usually either rectal or oesophageal) or have attempted to use poor surrogate measures of brain temperature such as tympanic temperature (Ansley et al., 2008). Mathematical modeling data suggests that superficial cooling of the brain may be possible (Zhu, 2000); however, clinical data have failed to identify a reduction in brain temperature following external cooling of the head and neck regions (Shiraki et al., 1988). These data suggest that it is probable that brain temperature was unaffected by the collar and cannot explain the improvements in performance observed in the present study or those reported previously. Central concentrations and ratios of, dopamine, noradrenaline and serotonin appear to be integral regulators of exercise performance in elevated ambient temperatures (Meeusen and Roelands, 2010); however, direct measurement of human cerebral concentrations during exercise is not possible and so peripheral concentrations are often used. One of the most commonly used indirect markers of central neurotransmission is prolactin (Chandler and Blair, 1980). Prolactin has been proposed as a peripheral marker of central fatigue in hot environments because its release is inhibited by dopaminergic, and stimulated by serotonergic, activity (Freeman et al., 2000); however, care when using prolactin as a marker is required because central serotonergic manipulation via nutritional or pharmacological interventions fails to alter the prolactin response to exercise (Strachan et al., 2004). Facial cooling during exercise can reduce the concentrations of prolactin (Brisson et al., 1987; Ansley et al., 2008); however, neck-cooling during prolonged exercise does not seem to effect concentrations (Tyler et al., 2010; Tyler and Sunderland, 2011b). The interaction effect, in the present study, suggests a lower prolactin concentration during the latter stages of exercise and this may suggest lower central fatigue during the collar cold trial, and potentially explains the increased power output observed during the latter sprints. Further research is clearly warranted to further investigate the relationship between neck cooling and central fatigue.

Peripheral concentrations of cortisol, which is often referred to as a “stress hormone,” were also unaffected by the cooling collar which is in line with previous data suggesting that cooling the neck may not improve performance sufficiently to alter the previously reported relationship between cortisol concentrations and exercise intensity (Fragala et al., 2011). It is worth noting that despite a lack of statistical significance cortisol concentrations were ~26% higher than baseline following the 1st set with the collar and ~20% lower than baseline without the collar at the same time point and this offers tentative support for data previously reported showing that cooling the neck has the potential to increase cortisol concentrations (Tyler and Sunderland, 2011b). Tyler and Sunderland (2011b) reported that the increases in cortisol were more pronounced when the neck was cooled during a pre-loaded time-trial from the start (+149 ± 193 nmol^.^l^−1^) than when no cooling was provided (+17 ± 202 nmol^.^l^−1^) or the cooling collar was replaced at regular intervals (+73 ± 133 nmol^.^l^−1^). In combination with the present data, these data highlight the variability observed in the cortisol response to exercise but also suggest that neck cooling may improve performance despite elevations in cortisol concentrations.

A further potential explanation for the improved repeated sprint performance, with neck cooling, could be the attenuation of the reduction in cerebral blood flow observed during exercise in the heat (Bain et al., 2015). As body temperature increases, during exercise in the heat, cerebral blood flow decreases resulting in cerebral hypoperfusion due to hyperthermia-induced hyperventilation (Rasmussen et al., 2006; Bain et al., 2015). This cerebral hypoperfusion may be an explanatory factor for the reduction in exercise performance in the heat. *In vitro* studies have demonstrated that heating carotid artery strips results in vasoconstriction (Mustafa et al., 2004) and conversely cooling results in carotid artery dilation (Mustafa and Thulesius, 2002). Therefore, neck cooling could result in carotid artery dilation, attenuation of cerebral hypoperfusion and thus enhanced performance. Clearly, this potential mechanism requires further investigation.

Repeated sprint performance was improved by neck-cooling in the current investigation, with decreased RPE but without significant alterations in thermal sensation. The lower relative RPE is in contrast with comparable steady state running (Tyler et al., 2010; Tyler and Sunderland, 2011a,b), while the lack of impact on thermal sensation is in line with some (Tyler and Sunderland, 2011a,b) but not all (Tyler et al., 2010; Lee et al., 2014) previous data. Non-cooling data have suggested that exercise in the heat can be improved at the cortical level if sensations are altered pharmacologically (Meeusen and Roelands, 2010). The decrease in RPE and lack of difference in thermal sensation could be interpreted as “recalibration” or “dampening” of the sensory cues which are combined to form this perceptual rating because more work was completed for the same, or lower, rating. The lower RPE and concomitant “dampening” of the sensory cues may contribute to the enhanced power output in the latter sprints because recent data suggests that self-paced high-intensity exercise is actively regulated in part by RPE (de Koning et al., 2011). Neck-cooling during exercise in the heat can dampen the level of thermal strain detected- allowing individuals to exercise for longer and tolerate higher levels of thermoregulatory and cardiovascular strain at the same levels of self-reported exertion and thermal state (Tyler and Sunderland, 2011a). Unsurprisingly, thermal sensation of the neck was significantly reduced via the application of the collar; however, despite the alliesthesial thermosensitivity of this region (Cotter and Taylor, 2005), this did not affect whole body thermal sensation.

The increase in power output observed when wearing the collar could be the result of a placebo effect and this cannot be ruled out. However, previous research has demonstrated that running performance is unchanged compared with no collar when wearing the collar uncooled (Tyler et al., 2010). Furthermore, some participants have reported finding the collar uncomfortable; despite this their performance is still improved. Clearly therefore, neck cooling could be advantageous for team sport athletes, both at recreational and elite level, in matches and training but consideration should be given to the ergonomic properties of the cooling used. For all intermittent sports neck cooling could be used during training. In competitive matches, for football and rugby union, neck cooling can take place throughout the “warm-up” and half-time break and in field hockey, ice hockey, and rugby league where continuous substitutions are permitted, neck cooling can be applied during “bench” time, which can be up to half the total match time. Neck cooling is also regularly utilized by tennis players during the breaks between games and sets. The small sample size of the present study is a limitation, but performance was statistically improved. Further, research is warranted with a larger sample size to further elucidate the mechanisms for the performance enhancement.

The present investigation is the first to show that cooling the neck during exercise can improve repeated sprint performance in the heat without altering the physiological or hormonal responses to repeated sprinting or a soccer-specific intermittent treadmill protocol. Neck-cooling can improve the subjective RPE and thermal comfort at the site of cooling and this localized improvement in thermal comfort may improve performance by masking the thermal strain of the body. The data from the current study compliments previous exercise and cognitive performance data and suggests that neck-cooling is a practical and effective intervention for the enhancement of intermittent, team-sport activity.

## Author contributions

CS designed the study with assistance from RS and BE. CS, RS, and BE completed the data collection. CS and CT completed the data analysis and manuscript preparation.

### Conflict of interest statement

The authors declare that the research was conducted in the absence of any commercial or financial relationships that could be construed as a potential conflict of interest.
